# Biased and Inflexible Interpretations of Social Situations Predict Affect Intensity and Variability in Children and Adolescents

**DOI:** 10.1007/s10802-025-01371-5

**Published:** 2025-09-04

**Authors:** Nicola Hohensee, Michael V. Bronstein, Jonas Everaert, Reut Zabag, Jutta Joormann, Reuma Gadassi-Polack

**Affiliations:** 1 Faculty of Sports and Psychology, University of Münster, Münster, Germany; 2 Yale Child Study Center, Yale School of Medicine, Yale University, CT New Haven, USA; 3 Department of Psychiatry & Behavioral Sciences, Institute for Health Informatics, University of Minnesota, Minneapolis, MN USA; 4 Tilburg School of Social and Behavioral Sciences, Tilburg University, Tilburg, The Netherlands; 5 Research Group of Quantitative Psychology and Individual Differences, KU Leuven, Leuven, Belgium; 6 Department of Psychology, Yale University, New Haven, CT USA; 7 The Gonda Multidisciplinary Brain Research Center, Bar-Ilan University, Ramat Gan, Israel; 8 Department of Psychiatry, Yale School of Medicine, Yale University, CT New Haven, USA

**Keywords:** Interpretation bias, Interpretation inflexibility, Affect dynamics, Adolescence

## Abstract

Adolescence is a developmental period characterized by heightened emotionality. Past research indicated that the biased interpretation of social situations (i.e., interpretation bias) might contribute to these aberrant affect dynamics in adolescents’ daily life. Social situations are, however, constantly changing. Therefore, the ability to flexibly revise social interpretations (i.e., interpretation flexibility) might be equally important as interpretation biases for explaining affective outcomes during adolescence. The present study examined whether interpretation bias and inflexibility predicted two major affect dynamics features (i.e., mean and standard deviation) in children and adolescents. At baseline, *N* = 154 children and adolescents (*M* = 12.81 years; 48.70% female; 49.35% male; 1.95% non-binary; 72.08% White) completed a task assessing bias and inflexibility in interpreting social situations. Then, for 28 days, participants rated their positive and negative affect. Results showed that inflexibility in revising positive and negative interpretations in light of disconfirming evidence predicted higher intensity of negative affect. When controlling for interpretation inflexibility, positive interpretation bias predicted higher positive affect, whereas negative interpretation bias was associated with more variable negative affect. The results emphasize the predictive utility of interpretation biases and inflexibility for daily affective experiences in youths.

**Supplementary Information:**

The online version contains supplementary material available at 10.1007/s10802-025-01371-5.

## Introduction

 In daily life, we are frequently confronted with ambiguous social situations. The negatively biased interpretations of ambiguous social situations may promote intense emotional reactions and facilitate a cascade of maladaptive changes in emotions and behaviors (Hirsch et al., 2016; Wichers et al., 2015; Wisco, 2009), especially when we are insufficiently responsive to disconfirmatory evidence (Gadassi Polack et al., 2023; Kube & Rosenkrantz, 2020).

Adolescents encounter numerous novel, ambiguous social situations in addition to experiencing changes in affect intensity and variability (Maciejewski et al., 2015; Rapee et al., 2019; Reitsema et al., 2022). These features (i.e., social and affective variability) of adolescence are likely connected (e.g., van Roeckel et al., 2015). Inaccurate interpretation of social situations – as occurs due to negative interpretation bias or insufficient interpretation updating – may evoke strong emotional and behavioral reactions (Wichers et al., 2015). Additionally, as adolescents are likely to be particularly sensitive to peer feedback, their emotional reactions to social situations are prone to be heightened (Somerville, 2013). With this in mind, the present study examined whether the biased and inflexible interpretation of social situations in youths contribute to less regulated affect (e.g., Maciejewski et al., 2015; Reitsema et al., 2022).

### Affect Intensity and Variability as Two Central Features of Affect Dynamics

*Affect intensity* captures how strongly an individual experiences positive or negative affect on average and is usually measured via the average across several measurement points (Reitsema et al., 2022). Many internalizing disorders that have their typical onset in adolescence are characterized by changes in affect intensity, such as heightened negative mean affect or lower positive mean affect (Kessler et al., 2005). From a developmental perspective, past research has shown that adolescence (vs. childhood) is characterized by increases in negative affect intensity while positive affect intensity remains stable (see Reitsema et al., 2022 for a meta-analysis).

Importantly, individuals typically experience deviations from their average affect intensity in daily life. The range of fluctuations around an individual’s average is usually referred to as *affect variability* and is measured via the within-person standard deviation across several measurement occasions (Reitsema et al., 2022). A recent meta-analysis (including up to *N* = 8 samples per analysis) did not identify any normative changes in affect variability across adolescence, besides from increases in sadness variability (Reitsema et al., 2022). Increases in positive and negative affect variability are, however, associated with lower psychological well-being and predict future mental health problems in youth (e.g., Neumann et al., 2011; Silk et al., 2003), which may be explainable by greater emotion regulation difficulties.^1^

### Association between interpretation bias and affect as well as psychopathology

Several studies have shown that adolescents with elevated symptoms of depression and anxiety tend to habitually endorse more negative and less positive interpretations of ambiguous social situations (Haller et al., 2016; Lau & Waters, 2017; Leigh & Clark, 2018; Mobach et al., 2019; Oliver et al., 2019; Sfärlea et al., 2021; Stuijfzand et al., 2018). In addition, decreases in negative and increases in positive interpretations via interpretation bias modification training were found to be associated with decreases in youths’ anxiety symptoms (see Krebs et al., 2018 for a meta-analysis). Similarly, Grocott et al. (2023) showed that higher negative interpretation bias in adolescents predicted more intense negative affect and less intense positive affect four months later. Finally, negative affect intensity mediated the association between negative interpretation bias and future depressive symptoms (Grocott et al., 2023). Taken together, these findings indicate that interpretation bias is related to the intensity of affect and psychopathology, beyond mere correlation.

### Interpretation Bias Inflexibility and its Association with Affect and Psychopathology

Beyond interpretation biases, current research increasingly focuses on the ability to flexibly shift an interpretation of a social situation when new information becomes available (Everaert et al., 2018; Gadassi Polack et al., 2023a; Mehu & Scherer, 2015; Stange et al., 2017) instead of using static stimuli (e.g., pictures or vignettes; see Platt et al., 2017 for a systematic review). In adults, difficulties in revising negative interpretations (i.e., interpretation inflexibility) was consistently associated with higher levels of psychopathology, above and beyond the effects of interpretation biases (Deng et al., 2023a, b; Deng et al., 2022; Duda et al., 2024; Everaert et al., 2018; Everaert et al., 2021; Everaert et al., 2020; Kube & Rozenkrantz, 2020). Importantly, some studies found evidence for associations between difficulties to revise positive interpretations when receiving disconfirming negative information (i.e., positive interpretation inflexibility) and symptoms of psychopathology as well (Deng et al., 2023a, b; Duda et al., 2024; Kube & Rosenkrantz, 2020). To our knowledge, only two studies assessed interpretation biases and inflexibility as well as their association with psychopathology in youth: Hollowell and Ronald (2020) found that interpretation inflexibility was positively associated with the degree of hallucinations in a community sample of adolescents. Gadassi Polack, et al. (2023a) showed that interpretation biases and inflexibility were associated with depressive and social anxiety symptoms. Importantly, negative and positive interpretation inflexibility attenuated depressive symptom reactivity to social events (Gadassi Polack et al., 2023a), suggesting that social interpretation inflexibility may contribute to less regulated and more unstable affect.

### Research Gaps

Despite this progress, two important questions remain. First, more studies focusing on interpretation inflexibility, interpretation biases, and affect in adolescents are needed. Most studies have examined interpretations in relation to adolescents’ psychopathology symptoms. The link between interpretation bias and affect is important to understand because affect dynamics are early risk markers for psychopathology (e.g., Lougheed et al., 2025; Neumann et al., 2011). Understanding associations between interpretation bias, interpretation inflexibility, and affect could reveal potential risk markers for psychopathology and predictors for well-being in this critical developmental period (Lougheed et al., 2025). In this regard, recent research also considers more complex affect dynamics like the degree of affect variability in addition to affect intensity (Reitsema et al., 2022). To our knowledge, no study to date assessed the association between biased or inflexible interpretation and this specific feature of affect dynamics.

Second, past research on interpretation bias and flexibility mostly used cross-sectional study designs or focused on long-term outcomes (i.e., months and years). More studies using intensive longitudinal designs, i.e., ecological momentary assessment or daily diaries, are needed. Intensive longitudinal designs are optimal for capturing situational variations (Russell & Gajos, 2020), which are especially important to consider when we are interested in more fine-grained changes of affect in the daily life of adolescents. In order to address the two resulting gaps in the literature, we examined interpretation bias and inflexibility as predictors of negative and positive affect intensity and variability in children and adolescents using a daily diary approach.

### Hypotheses

Based on previous findings, we formulated the following hypotheses:

#### Associations between interpretation bias, inflexibility, and mean affect intensity (Hypothesis 1)

Both negative and positive interpretation inflexibility at baseline would predict higher negative and lower positive mean affect intensity over the diary period, when controlling for interpretation biases (hypothesis 1a). More negative and less positive interpretation bias at baseline predict higher negative and lower positive mean affect intensity over the diary period, when controlling for interpretation inflexibility (hypothesis 1b).

#### Associations between interpretation bias, inflexibility, and affect variability (Hypothesis 2)

Both negative and positive interpretation inflexibility at baseline would be associated with higher (positive and negative) affect variability over the diary period, when controlling for interpretation biases and mean affect intensity (hypothesis 2a). More negative and less positive interpretation bias at baseline would be associated with higher (positive and negative) affect variability over the diary period, when controlling for interpretation inflexibility and mean affect intensity (hypothesis 2b).

Due to past findings regarding age and gender differences in interpretation bias and inflexibility (Gadassi Polack et al., 2023a; Habicht et al., 2022; Moutsiana et al., 2013), tests of all hypotheses above were repeated to probe for the moderating effects of age and gender.

## Method

The present study was part of a larger research project on emotions and social experiences in children and adolescents (see registration ID https://osf.io/kjq6g for further details on recruitment plan and measures). All relevant study materials and data analysis code for the current study can be found on OSF (project ID https://osf.io/bcf62/). Data is available by request from the last author. The larger research project included three data waves (see Gadassi-Polack et al., 2024). We used data from the third data wave because the task measuring bias and inflexibility was included only in the third data wave. Only relevant measures are described.

### Participants and Procedure

All procedures were approved by the Yale Institutional Review Board and we met APA ethical standards in the treatment of our sample. Participants were recruited either based on participation in previous data waves of the study or via social media ads inviting children and adolescents to participate in a study on emotions and social experiences. Data collection took place between June 2021 and March 2022. To be included in the study, youth had to reside in Conneticut, be between 9 and 18 years old and had to have access to a web-enabled device.

Power calculations for the first data wave of the larger research project (Gadassi-Polack et al., 2021; see more details below) indicated sufficient power with a minimum compliance rate of 60%. Because during the first data wave, participants completed 21 (instead of 28) diaries, minimum compliance rate was met when at least 13 diaries were completed, which informed compensation rate and exclusion criteria. In order to offset potential attrition, the number of diaries was increased to 28 from the second data wave on. To stay consistent with exclusion criteria during the first data wave, we only included adolescents in our analysis who completed at least 13 out of 28 daily diaries. This decision was pre-registered (https://osf.io/kjq6g) and is in line with general recommendations for intensive longitudinal study designs (Trull & Ebner-Priemer, 2020).

Data collection began with an onboarding session with youths and their parents. During the onboarding session, participants gave written informed consent and assent after a detailed briefing about the study procedure. These participants first completed via Qualtrics the Adolescent Emotional BADE Task (Gadassi-Polack et al., 2023a). Then, participants received links to daily diaries regarding their emotions and social interactions over the course of 28 days. Links were received via e-mail around their bedtime (i.e., at 7, 8 or 9 pm) and were valid for 16 hours. Youth received $15 for the completion of the baseline session, an additional $10 if they completed less than 60% of the daily diaries, $50 if they completed at least 60%, and $70 if they completed at least 90% of the surveys.

In total, *N* = 181 children and adolescents participated in both the Adolescent Emotional BADE Task at baseline and the daily diary. We excluded 27 participants from further analyses: 11 participants were excluded because of technical problems when administering the BADE Task, 11 youths failed various attention checks (e.g., *“How many hours are there in a single day?”* [12/10/24/28]; *“What kind of animal are you?”* [human/robot/puppy/kitten]) or showed deviating response processes from the other participants (i.e., relatively high person-total correlation on certain baseline questionnaires and/or relatively low intra-individual variability in the Emotional BADE task; for more details (see Gadassi-Polack et al. 2023a), and 5 additional participants were excluded because they completed less than 13 daily diaries. Thus, the final sample included *N* = 154 participants with an average age of *M* = 12.81 years (*SD* = 2.60 years, range = 9–18 years). Seventy-five youths identified as cisgender female, 76 as cisgender male and three as non-binary. 72.07% of the participants identified as White, 5.84% Asian, 1.95% Black, and 16.88% other ethnicities. Adherence rates to the daily diaries were high with *M* = 25.42 completed diaries (*SD* = 3.39), resulting in 3915 observations in total.

### Power Analysis

The sample size was determined based on power calculations conducted for the larger research project (see Gadassi-Polack et al., 2021 for more details). Gadassi-Polack et al. (2021) based power calculations on data and intra-class correlations derived from the first 18 participants, using the PASS software (https://www.ncss.com/software/pass). To detect main effects of interest with a power of 80%, they aimed to recruit at least 120 participants.

### Measures

Descriptive statistics and intraclass correlation coefficients for all daily diary measures can be found in Table 1 of the supplementary material.

#### Baseline

##### Adolescent Emotional Bias Against Disconfirmatory Evidence (BADE) Task - Version 1 (Gadassi-Polack et al., 2023a)

In this task, youths rate ambiguous social scenarios consisting of three sequentially presented statements. After each statement, participants are asked to rate the plausibility of four interpretations of the scenario events on a 13-point Likert scale from 1 (“*poor*”) to 13 (“*excellent*”). The four interpretations can be grouped into three different categories: One *absurd* interpretation is consistently implausible, two *lure* interpretations are initially most plausible and become less plausible throughout the task, and one *true* interpretation is initially moderately plausible and becomes most plausible in by the end of the task. Therefore, optimal performance in the BADE task requires flexible adaptation of scenario interpretations over the course of the task, depending on the provided statements. The BADE task contains two types of scenarios: (1) scenarios where initially negative interpretations are disconfirmed by positive information (“*disconfirming-the-negative scenarios*”), and (2) scenarios where initially positive interpretations are disconfirmed by negative information (“*disconfirming-the-positive scenarios*”). See an example in the supplements. 

The adolescent version of the BADE task was developed and validated in a previous study, where metrics haven proven to be correlated with depressive and anxiety symptoms (Gadassi-Polack et al., 2023a). Due to issues related to task development, this version of the adolescent BADE (version 1) consisted of 6 scenarios of disconfirming-the-positive but only 4 scenarios of disconfirming-the-negative (for details see Gadassi-Polack et al., 2023a). These two categories were analyzed separately, as was done in previous work (Everaert et al., 2018, 2020, 2021). The adolescent BADE had a very good internal consistency with a McDonald’s Omega total of *ω* = 0.94 for *absurd* statements, *ω* = 0.92 for *lure* statements, and *ω* = 0.82 for *true* statements. To ensure that younger children understood the BADE task’s instructions in a similar way to older children, we examined the BADE response patterns only for children between nine and eleven years and showed that these patterns were largely identical in comparison to the whole sample (see Figs. 1 and 2 in the supplemental material).

We conducted two robust principal component analyses with plausibility ratings, separately for disconfirming-the-negative and disconfirming-the-positive scenarios (Robust PCA; Hubert et al., 2005). PCA results confirmed three components for both scenario types: One component represented interpretation inflexibility as indicated by high positive loadings on the final *lure* interpretations and strong negative loadings on the final *true* interpretations. The second component consisted of high positive loadings of all *lure* interpretations and therefore, depending on the scenario type, represented either positive or negative interpretation bias (e.g., when initially negative *lure* interpretations in disconfirming-the-negative scenarios were strongly endorsed throughout the task, the component represented negative interpretation bias). The third component was assessed by high positive loadings for all *true* interpretations and therefore, similar to the second component, represented either positive or negative interpretation bias (e.g., when initially negative *true* interpretations in disconfirming-the-positive scenarios were strongly endorsed throughout the task, the component represented negative interpretation bias). The final metrics for negative interpretation bias, positive interpretation bias, and interpretation inflexibility were derived by adding all average explanation ratings that loaded highly onto each principal component. Based on the component loadings, we decided to adjust the final scoring slightly from the preregistration (https://osf.io/6vxf9) and the adult BADE version (e.g., Everaert et al., 2020). More specifically, we did not include *absurd* interpretations in the scoring of interpretation inflexibility because (in contrast to adult samples) absurd statements did not load strongly on inflexibility components. In addition, we included True1 in the scoring of positive interpretation inflexibility because of its high average loadings on this component. Thus, the final scoring scheme was LureA3 + LureB3-True3 for negative interpretation inflexibility, LureA3 + LureB3 + True1-True3^2^ for positive interpretation inflexibility, True1 + True2 + True3 for positive interpretation bias (disconfirming-the-negative scenarios) and negative interpretation bias (disconfirming-the-positive scenarios), LureA1 + LureA2 + LureB1 + LureB2 for negative interpretation bias (disconfirming-the-negative scenarios) and positive interpretation bias (disconfirming-the-positive scenarios). See Gadassi-Polack et al. (2023a) for further details on the scoring procedure and more detailed PCA results.

#### Daily Diaries

##### Affect

Items for the assessment of daily affect were partly based on the Positive and Negative Affect Schedule for Children (PANAS-C; Laurent et al., 1999) and on affect items used in previous studies (Gadassi et al., 2014; Snir et al., 2015). Eleven items assessed current negative affect (i.e., sad, upset, stressed, mad, miserable, nervous, guilty, lonely, ashamed, embarrassed, and jealous) and nine items assessed current positive affect (i.e., happy, excited, relaxed, joyful, grateful, proud, loved, respected, and liked). Participants were requested to report to what degree they felt each emotion at the moment of filling out the diary, on a 5-point Likert scale from 1 (“*very slightly or not at all*”) to 5 (“*extremely*”). The between-person and within-person reliabilities for negative affect (0.89 and 0.80, respectively) and positive affect (0.91 and 0.82, respectively) were very good in the present study (calculated following Nezlek, 2017; Shrout & Lane, 2012). All used affect items have been proven to be sensitive to symptoms of depression, anxiety or emotional instability in the past (Gadassi et al., 2014; Laurent et al., 1999; Snir et al., 2015).

### Data Analysis

Data were analyzed using the statistic software R version 4.2.0 (R Core Team, 2022).

#### Mean Affect Intensity as Outcome Variable (Hypotheses 1a and 1b)

To examine the hypotheses addressing associations between interpretation bias and inflexibility at baseline and mean affect intensity in daily life, we fitted two sets of multilevel regression models (i.e., for negative and positive affect) using the R package “lme4” version 1.1–31 (Bates et al., 2015). Daily assessments of affect (level 1) were nested within individuals (level 2). Multilevel models provide more accurate estimates than general linear regression models, because they account for the hierarchical data structure. We included random intercepts for all participants. Daily mean affect intensity was predicted from grand-mean centered BADE metrics (i.e., positive/negative interpretation inflexibility, positive, and negative interpretation bias). Separate models were constructed for metrics from disconfirming-the negative and disconfirming-the-positive scenarios, resulting in four models in total. One exemplary model for negative affect as outcome variable and BADE metrics from disconfirming-the-negative scenarios is presented below:

*Y*_*ik*_ (mean of negative affect of person *i* on day *k*) = *β*_*0i*_ + *r*_*ik*_

*β*_*0i*_ = *γ*_*00*_ + *γ*_*01*_ (negative interpretation inflexibility) + *γ*_*02*_ (negative interpretation bias) + *γ*_*03*_ (positive interpretation bias) + *u*_*0i*_.

Effect sizes (Cohen’s *f*^2^) for all predictors of interest were calculated as described in Selya et al. (2012) with *f*^2^ ≥ 0.02, *f*^2^ ≥ 0.15, and *f*^2^ ≥ 0.35 representing small, medium, and large effect sizes (Cohen, 1988).

#### Affect Variability as Outcome Variable (Hypotheses 2a and 2b)

To examine the hypotheses addressing associations between interpretation bias and inflexibility and affect variability, we conducted two sets of multilevel regression models (i.e., for negative and positive affect). Daily assessments (level 1) were nested within youths (level 2). We included random intercepts and slopes for daily average affect intensity. Daily affect variability (operationalized as the standard deviation; Dejonckheere et al., 2019) was predicted from grand-mean centered BADE metrics (i.e., interpretation inflexibility, positive, and negative interpretation bias). As recommended (Dejonckheere et al., 2019), all models controlled for daily person-mean centered average affect intensity. Again, separate models were constructed for BADE metrics from disconfirming-the negative and disconfirming-the-positive scenarios, resulting in four models in total. One exemplary model for negative affect as outcome variable and BADE metrics from disconfirming-the-negative scenarios is again presented below:

*Y*_*ik*_ (standard deviation of negative affect of person *i* on day *k*) = *β*_*0i*_ + *β*_*1i*_ (mean of basic negative affect on day *k*) + *r*_*ik*_.

*β*_*0i*_ = *γ*_*00*_ + *γ*_*01*_ (negative interpretation inflexibility) + *γ*_*02*_ (negative interpretation bias) + *γ*_*03*_ (positive interpretation bias) + *u*_*0j*_.

*β*_*1i*_ = *γ*_*10*_ + *u*_*1j*_.

Effect sizes (Cohen’s *f*^2^) were again calculated following Selya et al. (2012).

#### Effects of Age, Gender, and Racial Identity

We ran all the previous models including either age, gender, or racial identity as moderator variables to assess whether these variables affect the relation between interpretation inflexibility and daily mean affect intensity as well as daily affect variability. The 1.95% (*n* = 3) of participants who identified as non-binary were excluded from the analyses regarding gender differences due to the insufficient sample size for a meaningful examination of this group. Because the effects of gender, age, and racial identity were largely non-significant, we present them only in the supplementary materials (see Tables 2, 3, 4, 5, 6 and 7).

#### Multiple Comparisons Correction

Due to the high number of conducted statistical models, we corrected our analyses for multiple comparisons to reduce type 1 error. All reported results are adjusted using false discovery rate correction (Benjamini & Hochberg, 1995).

## Results

### Descriptives

Zero-order correlations between study variables are presented in Table 1. Regarding direct effects of age and gender, girls (vs. boys) and older (vs. younger) youths reported higher negative affect variability (see Tables 5 and 6 in the supplements). In addition, younger (vs. older) youths reported lower mean positive affect (but only when including BADE metrics from disconfirming-the-negative scenarios in the models; see Table 2 in the supplements). Thus, effects of age on mean positive affect were more fragile and less robust. Racial identity was not associated with mean affect or affect variability (see Tables 4 and 7 in the supplements).

**Table 1:** Pearson correlation matrix

Variable	1	2	3	4	5	6	7	8	9	10
1. NII (DNEG)	--									
2. NIB (DNEG)	0.09	--								
3. PIB (DNEG)	− 0.26*	− 0.27*	--							
4. PII (DPOS)	0.41*	− 0.40*	0.38*	--						
5. PIB (DPOS)	0.01	− 0.03	0.61*	0.42*	--					
6. NIB (DPOS)	− 0.03	0.61*	− 0.19*	− 0.37*	− 0.30*	--				
7. NA (*M*)	0.28*	0.06	− 0.08	0.15	− 0.04	0.02	--	− 0.44*	0.66*	− 0.02
8. PA (*M*)	− 0.03	− 0.07	0.29*	0.10	0.44*	− 0.13	− 0.11	--	− 0.31*	0.07*
9. NA (*SD*)	0.24*	0.25*	− 0.08	0.04	− 0.08	0.17*	0.73*	− 0.11	--	0.06*
10. PA (*SD*)	0.08	0.06	0.01	0.00	− 0.04	− 0.10	− 0.01	− 0.06	0.15	--

Within-person correlations are shown above the diagonal and between-person correlations are shown below the diagonal. DNEG = disconfirming the negative scenarios; DPOS = disconfirming the positive scenarios; NII = negative interpretation inflexibility; NIB = negative interpretation bias; PIB = positive interpretation bias; PII = positive interpretation inflexibility; NA = negative affect; PA = positive affect. * *p* <.05

### Associations between Interpretation Bias, Inflexibility, and Mean Affect Intensity (Hypothesis 1)

Estimates for all multilevel models are reported in Table 2.

**Table 2:** Estimates for multilevel regression models predicting daily mean affect intensity (*n* = 3915 observations)

	Estimate	df	t-value	*p*-value	0.95 CI	*p*_fdr_-value	RE (SD)
	DISCONFIRMING-THE-NEGATIVE SCENARIOS						
A. Negative Affect (conditional R^2^ = 0.62)							
**Intercept**	**1.51**	**149.78**	**37.48**	**< 0.001**	**1.44**,** 1.59**	**< 0.001**	0.50
**Negative Interpretation Inflexibility**	**0.14**	**150.03**	**3.40**	**< 0.001**	**0.06**,** 0.22**	**0.003**	
Negative Interpretation Bias	0.02	149.63	0.42	0.676	−0.07, 0.10	0.748	
Positive Interpretation Bias	0.00	149.82	0.04	0.971	−0.08, 0.09	0.971	
B. Positive Affect (conditional R^2^ = 0.66)							
**Intercept**	**2.93**	**150.00**	**45.59**	**< 0.001**	**2.80**,** 3.05**	**< 0.001**	0.79
Negative Interpretation Inflexibility	0.04	150.21	0.63	0.528	−0.09, 0.17	0.670	
Negative Interpretation Bias	0.01	149.87	0.11	0.912	−0.12, 0.14	0.930	
**Positive Interpretation Bias**	**0.25**	**150.03**	**3.70**	**< 0.001**	**0.12**,** 0.39**	**0.001**	
	DISCONFIRMING-THE-POSITIVE SCENARIOS						
C. Negative Affect (conditional R^2^ = 0.62)							
**Intercept**	**1.51**	**149.76**	**36.66**	**< 0.001**	**1.43**,** 1.59**	**< 0.001**	0.51
**Positive Interpretation Inflexibility**	**0.11**	**149.82**	**2.39**	**0.018**	**0.02**,** 0.21**	**0.045**	
Negative Interpretation Bias	0.03	149.70	0.74	0.461	−0.06, 0.12	0.599	
Positive Interpretation Bias	−0.06	149.68	−1.21	0.227	−0.15, 0.03	0.393	
D. Positive Affect (conditional R^2^ = 0.66)							
**Intercept**	**2.92**	**150.02**	**48.76**	**< 0.001**	**2.81**,** 3.04**	**< 0.001**	0.73
Positive Interpretation Inflexibility	−0.09	150.08	−1.30	0.195	−0.22, 0.04	0.349	
Negative Interpretation Bias	−0.03	149.95	−0.39	0.697	−0.15, 0.10	0.755	
**Positive Interpretation Bias**	**0.39**	**149.94**	**5.87**	**< 0.001**	**0.26**,** 0.52**	**< 0.001**	

The models presented here do not include any covariates; please refer to Tables 2, 3, 4, 5, 6 and 7 in the supplements for models including age, gender, or racial identity; significant estimates are written in bold font; Satterthwaite’s method was used for computing the degrees of freedom and t-statistics. RE = random effect; fdr = false discovery rate correction

#### Negative Affect

##### Hypothesis 1a

- In line with our hypothesis, participants with higher negative (*f*^2^ = 0.04) as well as positive (*f*^2^ = 0.02) interpretation inflexibility reported higher mean levels of negative affect.

##### Hypothesis 1b

- Unexpectedly, effects of positive and negative interpretation bias were non-significant for both, disconfirming-the-negative scenarios (all *f*^2^ < 0.01; all *p* > .05) and disconfirming-the-positive scenarios (all *f*^2^ < 0.01; all *p* > .05).

#### Positive Affect

##### Hypothesis 1a

- Neither negative nor positive interpretation inflexibility significantly predicted positive affect (all *f*^2^ < 0.01; all *p* > .05). These findings were not in line with our hypothesis.

##### Hypothesis 1b

- Higher levels of positive interpretation bias predicted higher mean levels of positive affect across disconfirming-the-negative (*f*^2^ = 0.06) and disconfirming-the-positive (*f*^2^ = 0.14) scenarios. These findings were in line with our hypothesis. Regarding negative interpretation bias, unexpectedly, neither associations with positive affect across disconfirming-the-negative (*f*^2^ < 0.01; *p* > .05) nor disconfirming-the-positive (*f*^2^ < 0.01; *p* > .05) scenarios were significant.

### Associations between Interpretation Bias, Inflexibility, and Affect Variability (Hypothesis 2)

Estimates for all multilevel models (controlled for mean affect intensity) are reported in Table 3.

**Table 3:** Estimates for multilevel regression models predicting affect variability (operationalized as daily standard deviation; *n* = 3915 observations)

	Estimate	df	t-value	*p*-value	0.95 CI	*p*_fdr_-value	RE (SD)
	DISCONFIRMING-THE-NEGATIVE SCENARIOS						
A. Negative Affect (conditional R^2^ = 0.91)							
**Intercept**	**0.54**	**148.88**	**21.42**	**< 0.001**	**0.49**,** 0.59**	**< 0.001**	0.31
Negative Interpretation Inflexibility	0.04	151.39	1.87	0.063	−0.00, 0.08	0.143	
**Negative Interpretation Bias**	**0.07**	**149.63**	**3.32**	**0.001**	**0.03**,** 0.11**	**0.003**	
Positive Interpretation Bias	−0.01	153.41	−0.55	0.584	−0.05, 0.03	0.690	
**NA Mean**	**1.03**	**127.45**	**17.93**	**< 0.001**	**0.92**,** 1.15**	**< 0.001**	0.69
B. Positive Affect (conditional R^2^ = 0.62)							
**Intercept**	**0.85**	**150.08**	**36.10**	**< 0.001**	**0.80**,** 0.89**	**< 0.001**	0.29
Negative Interpretation Inflexibility	0.02	150.42	0.98	0.328	−0.02, 0.07	0.534	
Negative Interpretation Bias	0.02	149.88	0.78	0.436	−0.03, 0.07	0.598	
Positive Interpretation Bias	0.01	150.13	0.57	0.567	−0.04, 0.07	0.687	
PA Mean	0.02	122.55	0.81	0.417	−0.03, 0.08	0.598	0.33
	DISCONFIRMING-THE-POSITIVE SCENARIOS						
C. Negative Affect (conditional R^2^ = 0.91)							
**Intercept**	**0.54**	**149.24**	**20.65**	**< 0.001**	**0.49**,** 0.59**	**< 0.001**	0.32
Positive Interpretation Inflexibility	0.01	149.93	0.49	0.627	−0.03, 0.06	0.708	
Negative Interpretation Bias	0.04	152.85	1.81	0.072	−0.00, 0.08	0.157	
Positive Interpretation Bias	−0.04	155.04	−1.58	0.117	−0.08, 0.01	0.234	
**NA Mean**	**1.03**	**126.80**	**17.90**	**< 0.001**	**0.92**,** 1.15**	**< 0.001**	0.69
D. Positive Affect (conditional R^2^ = 0.62)							
**Intercept**	**0.85**	**150.04**	**36.14**	**< 0.001**	**0.80**,** 0.89**	**< 0.001**	0.29
Positive Interpretation Inflexibility	−0.01	150.13	−0.20	0.839	−0.06, 0.05	0.872	
Negative Interpretation Bias	−0.04	149.95	−1.43	0.156	−0.09, 0.01	0.289	
Positive Interpretation Bias	−0.02	149.94	−0.82	0.413	−0.08, 0.03	0.598	
PA Mean	0.02	122.66	0.82	0.412	−0.03, 0.08	0.598	0.33

The models presented here do not include any covariates; please refer to Tables 2, 3, 4, 5, 6 and 7 in the supplements for models including age, gender, or racial identity; significant estimates are written in bold font; Satterthwaite’s method was used for computing the degrees of freedom and t-statistics. RE = random effect; NA = negative affect; PA = positive affect; fdr = false discovery rate correction

#### Negative Affect

##### Hypothesis 2a

- As expected, for disconfirming-the-negative scenarios, higher levels of negative interpretation inflexibility significantly predicted higher levels of negative affect variability. However, when mean affect intensity was controlled and false discovery rate correction was applied, the association for negative affect variability became non-significant (*f*^2^ = 0.01). For disconfirming-the-positive scenarios, levels of positive interpretation inflexibility did not predict levels of negative affect variability, which was not in line with our hypothesis (*f*^2^ < 0.01; *p* > .05). Taken together, no associations remained significant after false discovery rate correction and controlling for mean affect intensity.

##### Hypothesis 2b

- For disconfirming-the-negative scenarios, as expected, higher levels of negative interpretation bias predicted higher levels of variability in negative affect, even after controlling for mean affect intensity and applying false discovery rate correction (*f*^2^ = 0.03). We did not find, however, any significant predictive effect of positive interpretation bias (*f*^2^ < 0.01; *p* > .05). For disconfirming-the-positive scenarios, higher levels of negative interpretation bias predicted higher levels of variability in negative affect, in line with hypothesis 2b. After controlling for mean affect intensity and applying false discovery rate correction, however, the effect was no longer significant (*f*^2^ = 0.01). Surprisingly, associations with positive interpretation bias were non-significant across disconfirming-the-negative and disconfirming-the-positive scenarios (all *f*^2^ < 0.01; all *p* > .05). Taken together, only the association between negative interpretation bias (in initially negative scenarios) and negative affect variability remained significant.

#### Positive Affect

##### Hypothesis 2a

- Not in line with our hypothesis, effects for negative (*f*^2^ < 0.01; *p* > .05) and positive (*f*^2^ < 0.01; *p* > .05) interpretation inflexibility were non-significant.

##### Hypothesis 2b

- Similarly, all effects for negative and positive interpretation bias were non-significant across disconfirming-the-negative scenarios (all *f*^2^ < 0.01; all *p* > .05) and disconfirming-the-positive scenarios (all *f*^2^ < 0.01; all *p* > .05).

## Discussion

We provide novel and nuanced understanding about the role of biased and inflexible interpretations of social situations in adolescents’ affect intensity and variability, which are important risk markers for psychopathology. To our knowledge, this is the first study assessing the role of biased and inflexible interpretations in negative and positive affect dynamics during this critical developmental period. Our results show that interpretation bias and inflexibility have differential predictive value for affect dynamics in youths’ daily life. As expected, higher interpretation inflexibility predicted higher levels of negative affect, and higher positive interpretation bias predicted higher levels of positive affect. Additionally, negative interpretation bias predicted negative affect variability.

### Gender and Age Differences in Affect Dynamics

We did find a consistent pattern of age and gender differences for negative affect variability. Older adolescents reported higher negative affect variability in our sample, which is in line with research showing that affect variability especially during early adolescence increases (e.g., Larson et al., 2002). A recent meta-analysis, however, only identified significant increases in sadness variability throughout adolescence (Reitsema et al., 2022). In addition, similar to previous work, negative affect variability was higher in girls (Larson & Lampman-Petraitis, 1989; Weinstein & Mermelstein, 2013). Given the association between low emotional well-being and higher affect variability (e.g., Neumann et al., 2011), these results emphasize heightened psychological vulnerability during adolescence, especially for girls.

### Interpretation Inflexibility and Positive Interpretation Bias are Robustly Associated with Affect Intensity

In line with our hypotheses, the results showed that higher negative as well as positive interpretation inflexibility (i.e., difficulties in updating negative and positive interpretations of social situations in light of disconfirmatory evidence) were associated with more intense negative affect. Our results go beyond previous investigations that established links between negative interpretation bias and more intense overall negative affect (Grocott et al., 2023), because we examined the ability to revise initial interpretations in light of new information in addition to examining interpretation bias. Importantly, we showed that interpretation inflexibility holds greater predictive utility for negative affect intensity than interpretation biases. Negative (vs. positive) affect intensity holds high dispositional etiology (Diener & Emmons, 1984) and mean level differences in affect intensity are still the most robust predictor for emotional well-being and internalizing problems among all affect dynamic indices (Dejonckheere et al., 2019). Especially during adolescence, heightened negative affect intensity may be an expression of some adolescents being generally more emotionally reactive to (newly experienced) social situations than others (Somerville, 2016). Thus, our findings may indicate that the ability to revise interpretations in these social situations is more important for mean level differences in emotional well-being than the degree of bias in the initial interpretation. If future studies support this assumption, we should foster generally more flexible interpretation of social situations in adolescents to increase resilience and reduce risk for affective disturbances characteristic for internalizing disorders (Everaert et al., 2021; Gadassi Polack et al., 2023a; Grocott et al., 2023; Kashdan & Rottenberg, 2010; Mehu & Scherer, 2015; Stange et al., 2017).

In line with expectations, higher positive interpretation bias was associated with higher intensity of positive affect. This finding is in line with past research in adults and adolescents emphasizing the beneficial effects of a stronger positive interpretation bias, e.g., for decreases in psychopathology or enhanced resilience (Bean et al., 2023; Romano et al., 2020; Telman et al., 2013). Importantly, positive (vs. negative) interpretation bias is often neglected in research (Gadassi Polack et al., 2023b). This is concerning given that positive affect can facilitate prospective self-esteem, life satisfaction, and have a protective role against stressful life events in this critical developmental period (Coffey & Warren, 2020; Gilbert, 2012). These findings indicate that it may be worthwhile to strengthen adolescent’s positive interpretation biases in clinical interventions.

### Negative Interpretation Bias is most Robustly Associated with Affect Variability

As expected, adolescents with higher negative interpretation bias reported more variable negative affect. Given that more variable negative affect is associated with future mental health problems in adolescents (e.g., Neumann et al., 2011), one possible explanation for the link between interpretation bias and affect variability might be that youths who habitually interpret social situations more negatively have more difficulties in regulating the resulting emotions, which leads in turn to a less regulated, i.e., more variable, negative affect (e.g., Everaert et al., 2020; Gadassi Polack et al., 2023a). Future studies should examine possible mechanisms of associations between negative interpretation biases and affect dynamics (e.g., ineffective emotion regulation behavior) during adolescence more closely. The fact that negative interpretation bias (but not interpretation inflexibility) was linked to affect variability may also indicate that the initial bias in social situations is more relevant for context-dependent fluctuations in affect than the ability to revise interpretations (Reitsema et al., 2022). In contrast, interpretation inflexibility excelled interpretation bias in the prediction of more stable differences in affect intensity. One may speculate that these result patterns point towards different (more state vs. trait-based) operating mechanisms of interpretation bias and inflexibility but future research is needed to further explore this possibility.

Previous work has established links between negative interpretation biases in adolescents and depressive as well as anxiety symptoms (Haller et al., 2016; Lau & Waters, 2017; Leigh & Clark, 2018; Mobach et al., 2019; Oliver et al., 2019; Sfärlea et al., 2021; Stuijfzand et al., 2018). To our knowledge, only one previous study investigated the association between youth’s interpretation bias and daily affect markers, namely intensity and instability (Grocott et al., 2023). Grocott et al. (2023) showed that negative interpretation bias is related to negative affect intensity (but not instability). We extended these findings by examining affect variability (operationalized as the standard deviation) as an additional daily affect marker. Our findings underline that *how much* an adolescent’s negative affect fluctuates around their individual average (which may be an expression of more context-dependent emotion regulation processes) holds meaningful information beyond more stable differences in affect intensity.

The association between negative interpretation bias and negative affect variability was, however, only significant when the situation was generally negative to begin with. This effect was replicated when the situation was initially positive, however it did not survive controlling for mean affect. This emphasizes again the importance of considering mean affect as covariate when investigating more complex affect dynamics such as affect variability (Dejonckheere et al., 2019). In addition, affect variability was derived from daily diary assessments, which are not ideal in terms of time-resolution to capture variability in affect, which fluctuates significantly on a sub-daily basis (Shiffman et al., 2008). Future studies using even more intensive longitudinal designs (i.e., with assessments several times per day) are needed.

### Strengths and Limitations

The present study is, to our knowledge, the first to combine an objective, structured task assessing interpretation bias and inflexibility with a daily diary capturing affect dynamics in a large sample of children and adolescents. We thereby considered positive and negative valence, in regard to cognitive biases (i.e., negative and positive bias, negative and positive interpretation inflexibility) and affect (i.e., negative and positive affect intensity, negative and positive affect variability). Using an intensive longitudinal design enabled us additonally to include a large total number of 3915 observations in our analyses.

The present study also has some limitations that need to be acknowledged and might potentially explain some of our non-significant findings. The present investigation was conducted in a community sample, which is why the intensity and variability of negative affect were low compared to clinical samples (e.g., Nelson et al., 2020). This may have limited our power to detect small effects. Relatedly, we have a relatively homogenous sample regarding racial identity and little information about the socioeconomic status of our participants. To address these restrictions in generalizability, future studies assessing this missing information and using more diverse as well as clinical samples are needed.

## Conclusions

The current study holds significant implications for understanding the early markers of psychopathology in adolescence, a crucial period marked by the typical onset of many mental disorders (Kessler et al., 2005). Specifically, this study investigated the relationships between key cognitive and emotional risk factors, namely biased and inflexible interpretations and affect dynamics. Our results showed that difficulties in updating interpretations to social situations predicts more intense negative affect, whereas higher positive interpretation bias predicts more intense positive affect, and higher negative interpretation bias predicts more variable negative affect. We can draw several clinical implications from this work. While most interventions focus on interpretation biases, our study suggests that additionally fostering more flexible interpretation of social situations in adolescents may enhance resilience against mental health issues. Particularly among adolescents who exhibit heightened emotional reactivity, it may be important to address social interpretation styles. In addition, understanding that interpretation inflexibility and biases contribute to affect intensity and variability can help in the early identification of children and adolescents at risk of developing mental health problems. Educators and mental health practitioners can screen for these cognitive risk markers and provide early preventive interventions. Thus, directing attention towards both, interpretation bias and inflexibility may potentially lead to more effective prevention and intervention strategies for mental health problems in the future.

## Supplementary Information

Below is the link to the electronic supplementary material.

Supplementary Material 1

## Data Availability

All relevant study materials and data analysis code for the current study can be found on OSF (project ID https://osf.io/bcf62/). Data is available by request from the last author.

## Footnotes

1 We acknowledge that additional measures for affect dynamics were used in the past (e.g., emotional instability, inertia). Please refer to the supplements for a statement on alternative measures.

2 Our main results did not change when we excluded True1 from the scoring of positive interpretation inflexibility.
