# Supplementary material for: Biased and Inflexible Interpretations of Social Situations Predict Affect Intensity and Variability in Children and Adolescents
Source: Res Child Adolesc Psychopathol. 2025 Sep 4;53(10):1567–79. doi: 10.1007/s10802-025-01371-5 (PMC12521329; doi:10.1007/s10802-025-01371-5)
Supplement: Supplementary file 1 — Supplementary Material 1 [file 10802_2025_1371_MOESM1_ESM.docx]

**Supplements**

**Table 1**

*Descriptive statistics for BADE and daily affect measures*

|  | *M* | *SD_between_* | *SD_within_* | ICC |
| --- | --- | --- | --- | --- |
| Negative Interpretation Inflexibility (DNEG) | -0.03 | 0.59 | NA | NA |
| Negative Interpretation Bias (DNEG) | -0.03 | 0.87 | NA | NA |
| Positive Interpretation Bias (DNEG) | -0.01 | 0.55 | NA | NA |
| Positive Interpretation Inflexibility (DPOS) | -0.02 | 0.45 | NA | NA |
| Positive Interpretation Bias (DPOS) | 0.01 | 0.63 | NA | NA |
| Negative Interpretation Bias (DPOS) | -0.03 | 0.37 | NA | NA |
| Negative Affect | 1.51 | 0.52 | 0.54 | 0.62 |
| Positive Affect | 2.92 | 0.83 | 0.84 | 0.65 |

*Note.* DNEG = disconfirming the negative scenarios; DPOS = disconfirming the positive scenarios; ICC = intraclass correlation.

**Example for a BADE task scenario**

Find below an example for a *disconfirming-the-negative* scenario from the adolescent emotional BADE task.

**Situation**

*Some students from your school make faces as they board your bus and move as far away from you as possible.*

Please imagine yourself in this situation, as if you could see everything through your own eyes. Then, please tell us **how well** each explanation describes what is happening in the situation.

The students want privacy because they’re planning to take over the world. (*Absurd*)

The students want to make fun of you without you overhearing them. (*Lure A*)

The students want to avoid you because you are not cool enough for them. (*Lure B*)

The students want to play music without bothering anyone. (*True*)

**Situation**

*Some students from your school make faces as they board your bus and move as far away from you as possible.*

*You notice them laughing as they reach their seats.*

Please imagine yourself in this situation, as if you could see everything through your own eyes. Then, please tell us **how well** each explanation describes what is happening in the situation.

The students want privacy because they’re planning to take over the world. (*Absurd*)

The students want to make fun of you without you overhearing them. (*Lure A*)

The students want to avoid you because you are not cool enough for them. (*Lure B*)

The students want to play music without bothering anyone. (*True*)

**Situation**

*Some students from your school make faces as they board your bus and move as far away from you as possible.*

*You notice them laughing as they reach their seats.*

*They ask you to tell them if their music bothers you, then pull out a pair of speakers and start to play rock music.*

Please imagine yourself in this situation, as if you could see everything through your own eyes. Then, please tell us **how well** each explanation describes what is happening in the situation.

The students want privacy because they’re planning to take over the world. (*Absurd*)

The students want to make fun of you without you overhearing them. (*Lure A*)

The students want to avoid you because you are not cool enough for them. (*Lure B*)

The students want to play music without bothering anyone. (*True*)

**Additional analyses**

In the current study, we report analyses for mean affect intensity and affect variability. In the past, researchers used additional measures of affect dynamics (see Dejonckheere et al., 2019 for an overview). Therefore, we ran additional analyses with emotional instability (e.g., Trull et al., 2008), emotional inertia (e.g., Kuppens et al., 2010), and affective bipolarity (e.g., Dejonckheere et al., 2018) as outcome measures. The results were mostly non-significant and are therefore not reported here.

Dejonckheere, E., Mestdagh, M., Houben, M., Erbas, Y., Pe, M., Koval, P., ... & Kuppens, P. (2018). The bipolarity of affect and depressive symptoms. *Journal of Personality and Social Psychology, 114*(2), 323-341. https://doi.org/10.1037/pspp0000186

Dejonckheere, E., Mestdagh, M., Houben, M., Rutten, I., Sels, L., Kuppens, P., & Tuerlinckx, F. (2019). Complex affect dynamics add limited information to the prediction of psychological well-being. *Nature Human Behaviour*, *3*(5), 478–491. https://doi.org/10.1038/s41562-019-0555-0

Kuppens, P., Allen, N. B., & Sheeber, L. B. (2010). Emotional inertia and psychological maladjustment. *Psychological Science, 21*(7), 984-991. https://doi.org/10.1177/0956797610372634

Trull, T. J., Solhan, M. B., Tragesser, S. L., Jahng, S., Wood, P. K., Piasecki, T. M., & Watson, D. (2008). Affective instability: measuring a core feature of borderline personality disorder with ecological momentary assessment. *Journal of Abnormal Psychology, 117*(3), 647-661. https://doi.org/10.1037/a0012532

**Effects of age, gender, and racial identity**

One exemplary multilevel model for mean of negative affect as outcome variable and BADE metrics from disconfirming-the-negative scenarios is presented below:

*Y_ik_* (mean of negative affect of person *i* on day *k*) = *β_0i_* + *r_ik_*

*β_0i_* = *γ_00_* + *γ_01_* (negative interpretation inflexibility) + *γ_02_* (negative interpretation bias) + *γ_03_* (positive interpretation bias) + *γ_03_* (age/gender/racial identity) + *γ_04_* (age/gender/racial identity)(negative interpretation inflexibility) + *γ_05_* (age/gender/racial identity)(negative interpretation bias) + *γ_06_* (age/gender/racial identity)(positive interpretation bias) + *u_0i_*

One exemplary multilevel model for the standard deviation of negative affect as outcome variable and BADE metrics from disconfirming-the-negative scenarios is as follows:

*Y_ik_* (standard deviation of basic negative affect of person *i* on day *k*) = *β_0i_* + *β_1i_* (mean of basic negative affect on day *k*) + *r_ik_*

*β_0i_* = *γ_00_* + *γ_01_* (negative interpretation inflexibility) + *γ_02_* (negative interpretation bias) + *γ_03_* (positive interpretation bias) + *γ_03_* (age/gender/racial identity) + *γ_04_* (age/gender/racial identity)(negative interpretation inflexibility) + *γ_05_* (age/gender/racial identity)(negative interpretation bias) + *γ_06_* (age/gender/racial identity)(positive interpretation bias) + *u_0i_*

*β_1i_* = *γ_10_* + *u_1j_*

**Table 2**

*Estimates for multilevel regression models predicting daily mean affect and including age as moderator (n = 3915 observations)*

|  | | Estimate | *df* | *t*-*value* | *p-value* | .95 CI | RE (*SD*) |
| --- | --- | --- | --- | --- | --- | --- | --- |
|  | DISCONFIRMING-THE-NEGATIVE SCENARIOS | | | | | | |
| A. Negative Affect  **Intercept** | | **1.52** | **145.78** | **37.34** | **<.001** | **1.44; 1.60** | 0.50 |
| **NII** | | **0.14** | **146.11** | **3.24** | **.001** | **0.06; 0.22** |  |
| NIB | | 0.01 | 145.65 | 0.15 | .885 | -0.08; 0.09 |  |
| PIB | | -0.01 | 146.00 | -0.16 | .876 | -0.10; 0.08 |  |
| Age | | 0.07 | 145.59 | 1.66 | .099 | -0.01; 0.15 |  |
| NII:Age | | -0.03 | 145.94 | -0.67 | .502 | -0.10; 0.05 |  |
| NIB:Age | | -0.03 | 145.68 | -0.72 | .470 | -0.12; 0.05 |  |
| PIB:Age | | -0.01 | 145.76 | -0.32 | .748 | -0.09; 0.07 |  |
| B. Positive Affect  **Intercept** | | **2.91** | **146.09** | **45.50** | **<.001** | **2.79; 3.03** | 0.78 |
| NII | | 0.04 | 146.38 | 0.64 | .522 | -0.09; 0.17 |  |
| NIB | | 0.03 | 145.98 | 0.50 | .620 | -0.10; 0.16 |  |
| **PIB** | | **0.26** | **146.28** | **3.63** | **<.001** | **0.12; 0.40** |  |
| **Age** | | **-0.14** | **145.92** | **-2.19** | **.030** | **-0.27; -0.02** |  |
| NII:Age | | -0.05 | 146.23 | -0.87 | .387 | -0.16; 0.06 |  |
| NIB:Age | | 0.07 | 146.00 | 0.94 | .347 | -0.07; 0.20 |  |
| PIB:Age | | 0.03 | 146.07 | 0.47 | .638 | -0.09; 0.15 |  |
|  | DISCONFIRMING-THE-POSITIVE SCENARIOS | | | | | | |
| C. Negative Affect  **Intercept** | | **1.53** | **145.86** | **35.61** | **<.001** | **1.45; 1.61** | 0.51 |
| **PII** | | **0.11** | **145.76** | **2.35** | **.020** | **0.02; 0.21** |  |
| NIB | | 0.01 | 145.82 | 0.25 | .806 | -0.09; 0.11 |  |
| PIB | | -0.06 | 145.71 | -1.13 | .259 | -0.15; 0.04 |  |
| Age | | 0.06 | 145.71 | 1.46 | .146 | -0.02; 0.15 |  |
| PII:Age | | -0.04 | 145.92 | -1.04 | .300 | -0.13; 0.04 |  |
| NIB:Age | | -0.05 | 145.86 | -0.95 | .345 | -0.14; 0.05 |  |
| PIB:Age | | 0.03 | 145.55 | 0.49 | .626 | -0.07; 0.12 |  |
| D. Positive Affect  **Intercept** | | **2.92** | **146.17** | **46.84** | **<.001** | **2.80; 3.04** | 0.73 |
| PII | | -0.08 | 146.06 | -1.11 | .268 | -0.21; 0.06 |  |
| NIB | | 0.02 | 146.12 | 0.22 | .823 | -0.12; 0.16 |  |
| **PIB** | | **0.38** | **146.01** | **5.30** | **<.001** | **0.24; 0.52** |  |
| Age | | -0.08 | 146.02 | -1.23 | .222 | -0.20; 0.04 |  |
| PII:Age | | -0.11 | 146.23 | -1.74 | .085 | -0.22; 0.01 |  |
| NIB:Age | | 0.01 | 146.17 | 0.18 | .857 | -0.12; 0.14 |  |
| PIB:Age | | 0.07 | 145.85 | 0.95 | .346 | -0.07; 0.21 |  |

*Note.* NII = negative interpretation inflexibility; NIB = negative interpretation bias; PIB = positive interpretation bias; PII = positive interpretation inflexibility; RE = random effect. Significant estimates are written in bold font; Satterthwaite’s method was used for computing the degrees of freedom and t-statistics.

**Table 3**

*Estimates for multilevel regression models predicting daily mean affect and including gender as moderator (n = 3843 observations)*

|  | | Estimate | *df* | *t*-*value* | *p-value* | .95 CI | RE (*SD*) |
| --- | --- | --- | --- | --- | --- | --- | --- |
|  | DISCONFIRMING-THE-NEGATIVE SCENARIOS | | | | | | |
| A. Negative Affect  **Intercept** | | **1.46** | **143.01** | **25.19** | **<.001** | **1.35; 1.57** | 0.49 |
| **NII** | | **0.18** | **143.44** | **2.82** | **.005** | **0.06; 0.30** |  |
| NIB | | 0.02 | 142.74 | 0.41 | .683 | -0.09; 0.14 |  |
| PIB | | 0.01 | 142.99 | 0.15 | .878 | -0.12; 0.14 |  |
| Gender | | 0.10 | 142.84 | 1.18 | .242 | -0.06; 0.25 |  |
| NII:Gender | | -0.08 | 143.12 | -0.98 | .330 | -0.25; 0.08 |  |
| NIB:Gender | | -0.03 | 142.68 | -0.37 | .711 | -0.20; 0.13 |  |
| PIB:Gender | | -0.01 | 142.92 | -0.09 | .928 | -0.18; 0.16 |  |
| B. Positive Affect  **Intercept** | | **2.96** | **143.11** | **31.98** | **<.001** | **2.78; 3.14** | 0.79 |
| NII | | 0.15 | 143.47 | 1.42 | .159 | -0.05; 0.34 |  |
| NIB | | 0.06 | 142.88 | 0.67 | .503 | -0.12; 0.25 |  |
| PIB | | 0.19 | 143.09 | 1.86 | .066 | -0.01; 0.39 |  |
| Gender | | -0.09 | 142.97 | -0.67 | .505 | -0.34; 0.16 |  |
| NII:Gender | | -0.20 | 143.21 | -1.45 | .150 | -0.46; 0.07 |  |
| NIB:Gender | | -0.08 | 142.83 | -0.57 | .573 | -0.35; 0.19 |  |
| PIB:Gender | | 0.14 | 143.04 | 1.00 | .320 | -0.13; 0.41 |  |
|  | DISCONFIRMING-THE-POSITIVE SCENARIOS | | | | | | |
| C. Negative Affect  **Intercept** | | **1.46** | **143.05** | **24.40** | **<.001** | **1.34; 1.57** | 0.50 |
| **PII** | | **0.15** | **143.10** | **2.21** | **.029** | **0.02; 0.29** |  |
| NIB | | 0.05 | 143.19 | 0.87 | .389 | -0.06; 0.17 |  |
| PIB | | -0.05 | 142.90 | -0.70 | .483 | -0.17; 0.08 |  |
| Gender | | 0.10 | 142.89 | 1.16 | .248 | -0.06; 0.26 |  |
| **PII:Gender** | | -0.09 | 142.90 | -0.99 | .326 | -0.28; 0.09 |  |
| NIB:Gender | | -0.07 | 142.82 | -0.75 | .454 | -0.26; 0.12 |  |
| PIB:Gender | | -0.04 | 142.81 | -0.43 | .667 | -0.22; 0.14 |  |
| D. Positive Affect  **Intercept** | | **2.97** | **143.27** | **34.14** | **<.001** | **2.80; 3.14** | 0.73 |
| PII | | 0.01 | 143.32 | 0.08 | .936 | -0.19; 0.20 |  |
| NIB | | 0.11 | 143.41 | 1.28 | .201 | -0.06; 0.28 |  |
| **PIB** | | **0.32** | **143.11** | **3.35** | **.001** | **0.14; 0.50** |  |
| Gender | | -0.04 | 143.10 | -0.29 | .769 | -0.27; 0.20 |  |
| PII:Gender | | -0.16 | 143.12 | -1.14 | .254 | -0.43; 0.11 |  |
| **NIB:Gender** | | **-0.33** | **143.03** | **-2.31** | **.022** | **-0.61; -0.06** |  |
| PIB:Gender | | 0.10 | 143.02 | 0.73 | .465 | -0.17; 0.37 |  |

*Note.* Sample does not include participants who identified as non-binary. NII = negative interpretation inflexibility; NIB = negative interpretation bias; PIB = positive interpretation bias; PII = positive interpretation inflexibility; RE = random effect. Significant estimates are written in bold font; Satterthwaite’s method was used for computing the degrees of freedom and t-statistics.

**Table 4**

*Estimates for multilevel regression models predicting daily mean affect and including racial identity as moderator (n = 3915 observations)*

|  | | Estimate | *df* | *t*-*value* | *p-value* | .95 CI | RE (*SD*) |
| --- | --- | --- | --- | --- | --- | --- | --- |
|  | DISCONFIRMING-THE-NEGATIVE SCENARIOS | | | | | | |
| A. Negative Affect  **Intercept** | | **1.49** | **145.72** | **32.55** | **<.001** | **1.40; 1.58** | 0.50 |
| **NII** | | **0.16** | **146.26** | **3.35** | **.001** | **0.07; 0.26** |  |
| NIB | | 0.03 | 145.50 | 0.71 | .482 | -0.06; 0.12 |  |
| PIB | | 0.01 | 145.48 | 0.23 | .819 | -0.08; 0.10 |  |
| Race | | 0.05 | 145.72 | 1.09 | .276 | -0.04; 0.14 |  |
| NII:Race | | -0.02 | 146.26 | -0.48 | .631 | -0.12; 0.07 |  |
| NIB:Race | | -0.05 | 145.50 | -1.00 | .321 | -0.14; 0.04 |  |
| PIB:Race | | -0.04 | 145.48 | -0.79 | .432 | -0.13; 0.06 |  |
| B. Positive Affect  **Intercept** | | **2.90** | **145.96** | **39.76** | **<.001** | **2.76; 3.04** | 0.79 |
| NII | | 0.10 | 146.43 | 1.27 | .206 | -0.05; 0.25 |  |
| NIB | | 0.03 | 145.77 | 0.44 | .658 | -0.11; 0.18 |  |
| **PIB** | | **0.27** | **145.76** | **3.48** | **.001** | **0.12; 0.41** |  |
| Race | | 0.05 | 145.96 | 0.66 | .510 | -0.09; 0.19 |  |
| NII:Race | | -0.10 | 146.43 | -1.35 | .180 | -0.25; 0.04 |  |
| NIB:Race | | -0.04 | 145.77 | -0.59 | .558 | -0.19; 0.10 |  |
| PIB:Race | | -0.04 | 145.76 | -0.58 | .565 | -0.19; 0.10 |  |
|  | DISCONFIRMING-THE-POSITIVE SCENARIOS | | | | | | |
| C. Negative Affect  **Intercept** | | **1.49** | **145.69** | **31.61** | **<.001** | **1.40; 1.58** | 0.51 |
| **PII** | | **0.13** | **145.93** | **2.45** | **.016** | **0.03; 0.23** |  |
| NIB | | 0.05 | 145.78 | 1.02 | .326 | -0.05; 0.15 |  |
| PIB | | -0.07 | 145.65 | -1.38 | .170 | -0.17; 0.03 |  |
| Race | | 0.05 | 145.69 | 0.99 | .326 | -0.04; 0.14 |  |
| **PII:**Race | | -0.03 | 145.93 | -0.57 | .572 | -0.13; 0.07 |  |
| NIB:Race | | -0.04 | 145.78 | -0.75 | .455 | -0.14; 0.06 |  |
| PIB:Race | | 0.02 | 145.65 | 0.31 | .757 | -0.08; 0.12 |  |
| D. Positive Affect  **Intercept** | | **2.92** | **145.94** | **42.40** | **<.001** | **2.78; 3.05** | 0.74 |
| PII | | -0.08 | 146.19 | -0.99 | .322 | -0.23; 0.07 |  |
| NIB | | -0.02 | 146.04 | -0.27 | .790 | -0.16; 0.12 |  |
| **PIB** | | **0.38** | **145.90** | **4.98** | **<.001** | **0.23; 0.53** |  |
| Race | | 0.01 | 145.94 | 0.13 | .897 | -0.12; 0.14 |  |
| PII:Race | | -0.02 | 146.19 | -0.29 | .772 | -0.17; 0.13 |  |
| NIB:Race | | -0.01 | 146.04 | -0.13 | .898 | -0.15; 0.13 |  |
| PIB:Race | | 0.03 | 145.90 | 0.34 | .737 | -0.12; 0.17 |  |

*Note.* NII = negative interpretation inflexibility; NIB = negative interpretation bias; PIB = positive interpretation bias; PII = positive interpretation inflexibility; RE = random effect. Significant estimates are written in bold font; Satterthwaite’s method was used for computing the degrees of freedom and t-statistics.

**Table 5**

*Estimates for multilevel regression models predicting daily affect variability and including age as moderator (n = 3915 observations)*

|  | | Estimate | *df* | *t*-*value* | *p-value* | .95 CI | RE (*SD*) |
| --- | --- | --- | --- | --- | --- | --- | --- |
|  | DISCONFIRMING-THE-NEGATIVE SCENARIOS | | | | | | |
| A. Negative Affect  **Intercept** | | **0.54** | **146.91** | **21.94** | **<.001** | **0.50; 0.59** | 0.30 |
| NII | | 0.04 | 147.33 | 1.93 | .056 | -0.00; 0.08 |  |
| **NIB** | | **0.06** | **144.86** | **3.10** | **.002** | **0.02; 0.10** |  |
| PIB | | -0.01 | 148.57 | -0.43 | .669 | -0.05; 0.03 |  |
| **Age** | | **0.05** | **147.62** | **2.63** | **.010** | **0.01; 0.09** |  |
| **Mean NA** | | **1.03** | **127.12** | **17.94** | **<.001** | **0.92; 1.15** | 0.69 |
| NII:Age | | 0.00 | 144.21 | 0.23 | .820 | -0.03; 0.04 |  |
| NIB:Age | | 0.01 | 146.17 | 0.38 | .708 | -0.03; 0.05 |  |
| PIB:Age | | 0.01 | 147.88 | 0.41 | .681 | -0.03; 0.04 |  |
| B. Positive Affect  **Intercept** | | **0.84** | **146.00** | **35.91** | **<.001** | **0.80; 0.89** | 0.28 |
| NII | | 0.02 | 146.44 | 0.73 | .467 | -0.03; 0.07 |  |
| NIB | | 0.02 | 145.81 | 0.92 | .360 | -0.03; 0.07 |  |
| PIB | | 0.01 | 146.29 | 0.21 | .837 | -0.05; 0.06 |  |
| Age | | -0.03 | 145.71 | -1.35 | .179 | -0.08; 0.01 |  |
| Mean PA | | 0.02 | 122.38 | 0.80 | .428 | -0.03; 0.08 | 0.33 |
| NII:Age | | -0.00 | 146.19 | -0.15 | .885 | -0.04; 0.04 |  |
| NIB:Age | | 0.01 | 145.84 | 0.50 | .619 | -0.04; 0.06 |  |
| PIB:Age | | -0.04 | 145.95 | -1.53 | .129 | -0.08; 0.01 |  |
|  | DISCONFIRMING-THE-POSITIVE SCENARIOS | | | | | | |
| C. Negative Affect  **Intercept** | | **0.55** | **152.23** | **20.79** | **<.001** | **0.50; 0.60** | 0.32 |
| PII | | 0.01 | 146.12 | 0.43 | .671 | -0.03; 0.05 |  |
| NIB | | 0.02 | 145.39 | 1.00 | .319 | -0.02; 0.07 |  |
| PIB | | -0.03 | 147.32 | -1.32 | .187 | -0.08; 0.01 |  |
| **Age** | | **0.04** | **145.62** | **2.16** | **.032** | **0.01; 0.08** |  |
| **Mean NA** | | **1.03** | **126.51** | **17.90** | **<.001** | **0.92; 1.15** | 0.69 |
| PII:Age | | 0.00 | 147.25 | 0.16 | .877 | -0.04; 0.04 |  |
| NIB:Age | | -0.02 | 147.06 | -0.74 | .461 | -0.06; 0.03 |  |
| PIB:Age | | -0.00 | 150.44 | -0.07 | .947 | -0.05; 0.05 |  |
| D. Positive Affect  **Intercept** | | **0.84** | **146.18** | **34.34** | **<.001** | **0.79; 0.88** | 0.28 |
| PII | | -0.01 | 146.03 | -0.33 | .740 | -0.06; 0.04 |  |
| NIB | | -0.04 | 146.11 | -1.30 | .197 | -0.09; 0.02 |  |
| PIB | | -0.03 | 145.95 | -1.11 | .269 | -0.09; 0.03 |  |
| Age | | -0.03 | 145.96 | -1.27 | .205 | -0.08; 0.02 |  |
| Mean PA | | 0.02 | 122.63 | 0.82 | .413 | -0.03; 0.08 | 0.33 |
| PII:Age | | 0.02 | 146.25 | 1.00 | .318 | -0.02; 0.07 |  |
| NIB:Age | | 0.00 | 146.17 | 0.11 | .909 | -0.05; 0.06 |  |
| PIB:Age | | -0.04 | 145.72 | -1.44 | .151 | -0.10; 0.01 |  |

*Note.* NA = negative affect; PA = positive affect; NII = negative interpretation inflexibility; NIB = negative interpretation bias; PIB = positive interpretation bias; PII = positive interpretation inflexibility; RE = random effect. Significant estimates are written in bold font; Satterthwaite’s method was used for computing the degrees of freedom and t-statistics.

**Table 6**

*Estimates for multilevel regression models predicting daily affect variability and including gender as moderator (n = 3843 observations)*

|  | | Estimate | *df* | *t*-*value* | *p-value* | .95 CI | RE (*SD*) |
| --- | --- | --- | --- | --- | --- | --- | --- |
|  | DISCONFIRMING-THE-NEGATIVE SCENARIOS | | | | | | |
| A. Negative Affect  **Intercept** | | **0.48** | **178.75** | **15.64** | **<.001** | **0.42; 0.54** | 0.30 |
| **NII** | | **0.07** | **149.09** | **2.32** | **.022** | **0.01; 0.12** |  |
| NIB | | 0.05 | 143.97 | 1.75 | .082 | -0.01; 0.10 |  |
| PIB | | 0.01 | 148.13 | 0.34 | .738 | -0.05; 0.07 |  |
| **Gender** | | **0.12** | **143.90** | **3.21** | **.002** | **0.05; 0.19** |  |
| **Mean NA** | | **1.04** | **125.02** | **17.95** | **<.001** | **0.93; 1.16** | 0.69 |
| NII:Gender | | -0.05 | 144.35 | -1.22 | .226 | -0.12; 0.03 |  |
| NIB:Gender | | 0.02 | 140.81 | 0.51 | .608 | -0.05; 0.09 |  |
| PIB:Gender | | -0.04 | 145.76 | -0.88 | .382 | -0.11; 0.04 |  |
| B. Positive Affect  **Intercept** | | **0.83** | **143.25** | **24.66** | **<.001** | **0.77; 0.90** | 0.29 |
| NII | | 0.02 | 143.83 | 0.55 | .581 | -0.05; 0.09 |  |
| NIB | | -0.02 | 142.88 | -0.59 | .557 | -0.09; 0.05 |  |
| PIB | | -0.02 | 143.22 | -0.48 | .632 | -0.09; 0.06 |  |
| Gender | | 0.01 | 143.03 | 0.30 | .765 | -0.08; 0.11 |  |
| Mean PA | | 0.02 | 120.38 | 0.76 | .447 | -0.04; 0.08 | 0.34 |
| NII:Gender | | -0.01 | 143.40 | -0.15 | .878 | -0.10; 0.09 |  |
| NIB:Gender | | 0.08 | 142.80 | 1.61 | .109 | -0.02; 0.18 |  |
| PIB:Gender | | 0.07 | 143.13 | 1.32 | .190 | -0.03; 0.17 |  |
|  | DISCONFIRMING-THE-POSITIVE SCENARIOS | | | | | | |
| C. Negative Affect  **Intercept** | | **0.48** | **179.90** | **14.91** | **<.001** | **0.42; 0.54** | 0.31 |
| PII | | 0.04 | 148.43 | 1.32 | .189 | -0.02; 0.11 |  |
| NIB | | 0.03 | 148.82 | 1.13 | .258 | -0.02; 0.09 |  |
| PIB | | -0.03 | 150.82 | -0.91 | .363 | -0.09; 0.03 |  |
| **Gender** | | **0.11** | **144.42** | **2.82** | **.006** | **0.04; 0.19** |  |
| **Mean NA** | | **1.05** | **124.35** | **17.92** | **<.001** | **0.93; 1.16** | 0.69 |
| **PII:Gender** | | -0.06 | 143.71 | -1.31 | .192 | -0.14; 0.03 |  |
| NIB:Gender | | -0.03 | 143.32 | -0.64 | .522 | -0.12; 0.06 |  |
| PIB:Gender | | -0.01 | 146.63 | -0.22 | .827 | -0.09; 0.08 |  |
| D. Positive Affect  **Intercept** | | **0.83** | **143.37** | **23.93** | **<.001** | **0.76; 0.89** | 0.29 |
| PII | | -0.01 | 143.42 | -0.12 | .907 | -0.08; 0.07 |  |
| NIB | | -0.06 | 143.54 | -1.67 | .097 | -0.13; 0.01 |  |
| PIB | | -0.02 | 143.13 | -0.46 | .650 | -0.09; 0.06 |  |
| Gender | | 0.03 | 143.12 | 0.59 | .553 | -0.07; 0.12 |  |
| Mean PA | | 0.02 | 120.51 | 0.78 | .440 | -0.04; 0.08 | 0.34 |
| PII:Gender | | -0.01 | 143.14 | -0.15 | .881 | -0.11; 0.10 |  |
| NIB:Gender | | 0.04 | 143.02 | 0.67 | .501 | -0.07; 0.15 |  |
| PIB:Gender | | 0.01 | 143.01 | 0.20 | .846 | -0.10; 0.12 |  |

*Note.* Sample does not include participants who identified as non-binary. NA = negative affect; PA = positive affect; NII = negative interpretation inflexibility; NIB = negative interpretation bias; PIB = positive interpretation bias; PII = positive interpretation inflexibility; RE = random effect. Significant estimates are written in bold font; Satterthwaite’s method was used for computing the degrees of freedom and t-statistics.

**Table 7**

*Estimates for multilevel regression models predicting daily affect variability and including racial identity as moderator (n = 3915 observations)*

|  | | Estimate | *df* | *t*-*value* | *p-value* | .95 CI | RE (*SD*) |
| --- | --- | --- | --- | --- | --- | --- | --- |
|  | DISCONFIRMING-THE-NEGATIVE SCENARIOS | | | | | | |
| A. Negative Affect  **Intercept** | | **0.55** | **160.71** | **20.11** | **<.001** | **0.50; 0.61** | 0.31 |
| NII | | 0.03 | 153.60 | 1.28 | .204 | -0.02; 0.08 |  |
| **NIB** | | **0.06** | **146.43** | **2.85** | **.005** | **0.02; 0.11** |  |
| PIB | | -0.00 | 146.86 | -0.12 | .908 | -0.05; 0.04 |  |
| Race | | -0.02 | 150.73 | -0.84 | .401 | -0.06; 0.02 |  |
| **Mean NA** | | **1.03** | **127.50** | **17.94** | **<.001** | **0.92; 1.15** | 0.69 |
| NII:Race | | 0.01 | 153.57 | 0.42 | .675 | -0.04; 0.05 |  |
| NIB:Race | | -0.00 | 146.41 | -0.06 | .956 | -0.04; 0.04 |  |
| PIB:Race | | -0.02 | 146.87 | -0.87 | .385 | -0.06; 0.02 |  |
| B. Positive Affect  **Intercept** | | **0.84** | **146.00** | **31.42** | **<.001** | **0.79; 0.89** | 0.29 |
| NII | | 0.03 | 146.73 | 1.22 | .226 | -0.02; 0.09 |  |
| NIB | | 0.02 | 145.70 | 0.71 | .482 | -0.03; 0.07 |  |
| PIB | | 0.02 | 145.68 | 0.74 | .461 | -0.04; 0.08 |  |
| Race | | 0.01 | 146.00 | 0.36 | .722 | -0.04; 0.06 |  |
| Mean PA | | 0.02 | 122.50 | 0.81 | .420 | -0.03; 0.08 | 0.33 |
| NII:Race | | -0.02 | 146.73 | -0.80 | .424 | -0.08; 0.03 |  |
| NIB:Race | | 0.01 | 145.70 | 0.19 | .852 | -0.05; 0.06 |  |
| PIB:Race | | -0.01 | 145.68 | -0.42 | .676 | -0.07; 0.04 |  |
|  | DISCONFIRMING-THE-POSITIVE SCENARIOS | | | | | | |
| C. Negative Affect  **Intercept** | | **0.55** | **162.53** | **19.41** | **<.001** | **0.50; 0.61** | 0.33 |
| PII | | -0.00 | 147.21 | -0.17 | .866 | -0.05; 0.05 |  |
| NIB | | 0.04 | 151.50 | 1.55 | .124 | -0.01; 0.08 |  |
| PIB | | -0.02 | 148.44 | -0.85 | .396 | -0.07; 0.03 |  |
| Race | | -0.02 | 149.47 | -0.98 | .327 | -0.07; 0.02 |  |
| **Mean NA** | | **1.03** | **126.75** | **17.90** | **<.001** | **0.92; 1.15** | 0.69 |
| **PII:**Race | | 0.03 | 147.19 | 1.05 | .295 | -0.02; 0.08 |  |
| NIB:Race | | -0.00 | 151.47 | -0.08 | .935 | -0.05; 0.05 |  |
| PIB:Race | | -0.03 | 148.43 | -1.03 | .307 | -0.07; 0.02 |  |
| D. Positive Affect  **Intercept** | | **0.84** | **145.97** | **31.46** | **<.001** | **0.79; 0.89** | 0.29 |
| PII | | -0.01 | 146.30 | -0.24 | .811 | -0.07; 0.05 |  |
| NIB | | -0.04 | 146.08 | -1.25 | .212 | -0.09; 0.02 |  |
| PIB | | -0.02 | 145.90 | -0.56 | .576 | -0.08; 0.04 |  |
| Race | | 0.01 | 145.95 | 0.23 | .818 | -0.05; 0.06 |  |
| Mean PA | | 0.02 | 122.65 | 0.82 | .412 | -0.03; 0.08 | 0.33 |
| PII:Race | | 0.00 | 146.30 | 0.11 | .911 | -0.06; 0.06 |  |
| NIB:Race | | -0.00 | 146.08 | -0.13 | .898 | -0.06; 0.05 |  |
| PIB:Race | | -0.01 | 145.90 | -0.45 | .653 | -0.07; 0.04 |  |

*Note.* NA = negative affect; PA = positive affect; NII = negative interpretation inflexibility; NIB = negative interpretation bias; PIB = positive interpretation bias; PII = positive interpretation inflexibility; RE = random effect. Significant estimates are written in bold font; Satterthwaite’s method was used for computing the degrees of freedom and t-statistics.

**Figure 1**

*Scenario structure for disconfirming-the-positive scenarios (only youths between nine and eleven years)*


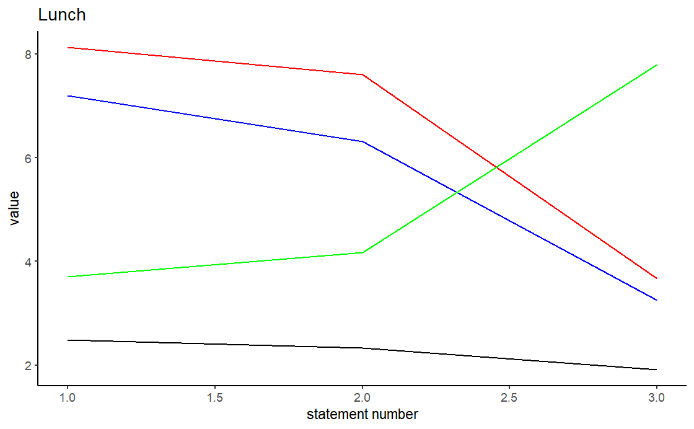

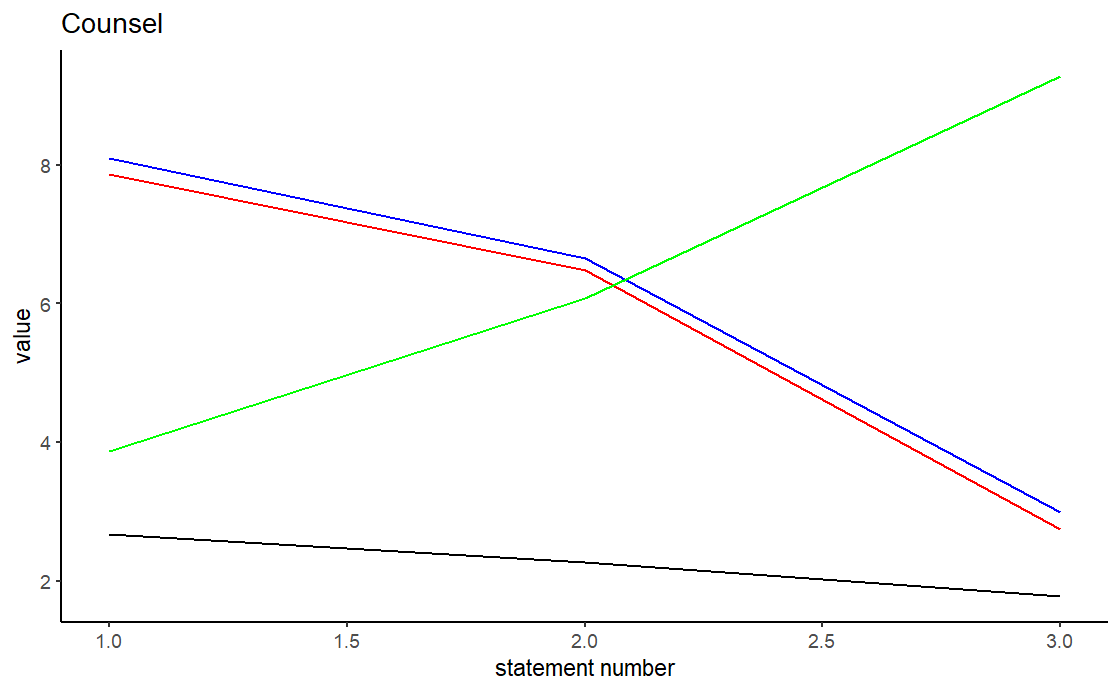


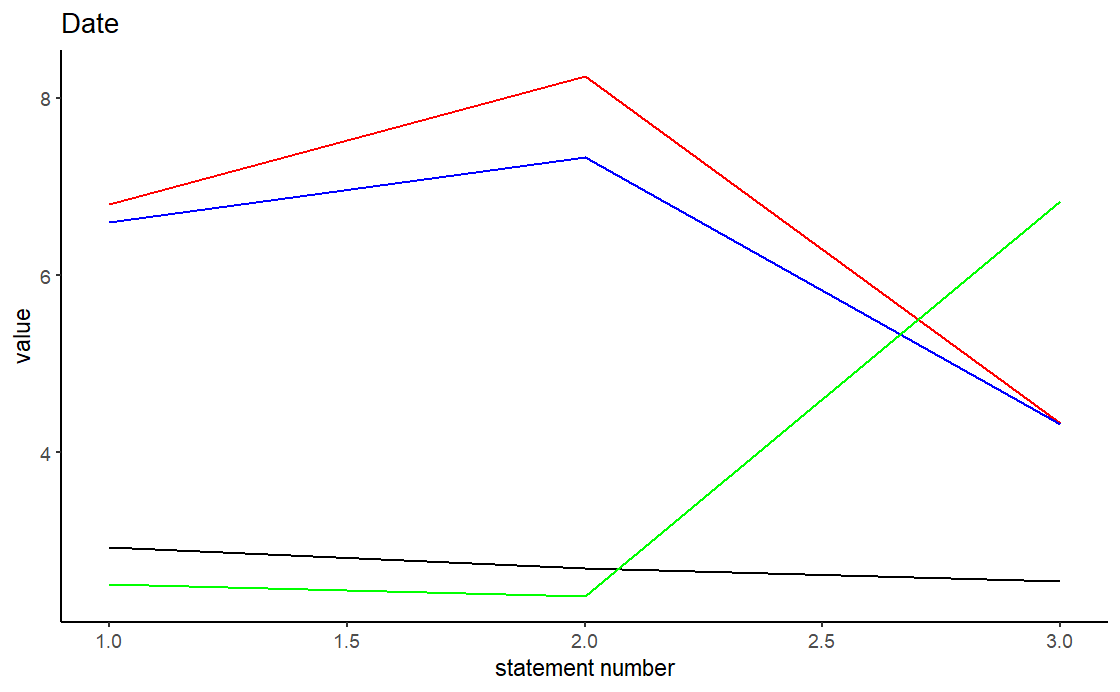


*Note.* The trend for average ratings for each explanation type are represented in a different line color. Green=True, Red/Blue=Lure, Black=Absurd. Scenario names are given at the top-left of each panel. Figures for the whole sample can be found in the supplements of Gadassi-Polack, Bronstein, et al. (2023).

**Figure 2**

*Scenario structure for disconfirming-the-negative scenarios (only youths between nine and eleven years)*


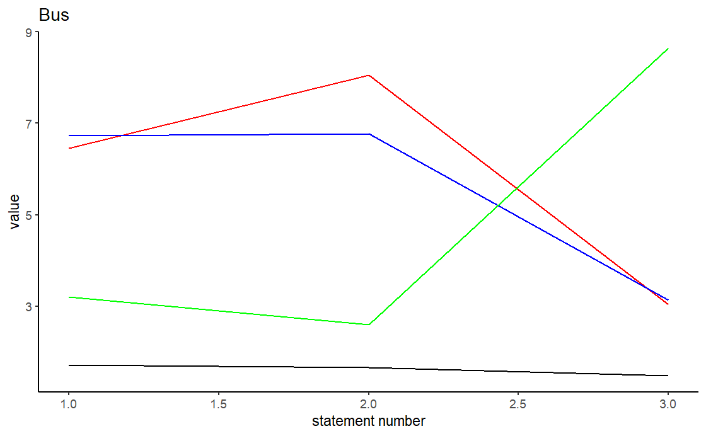

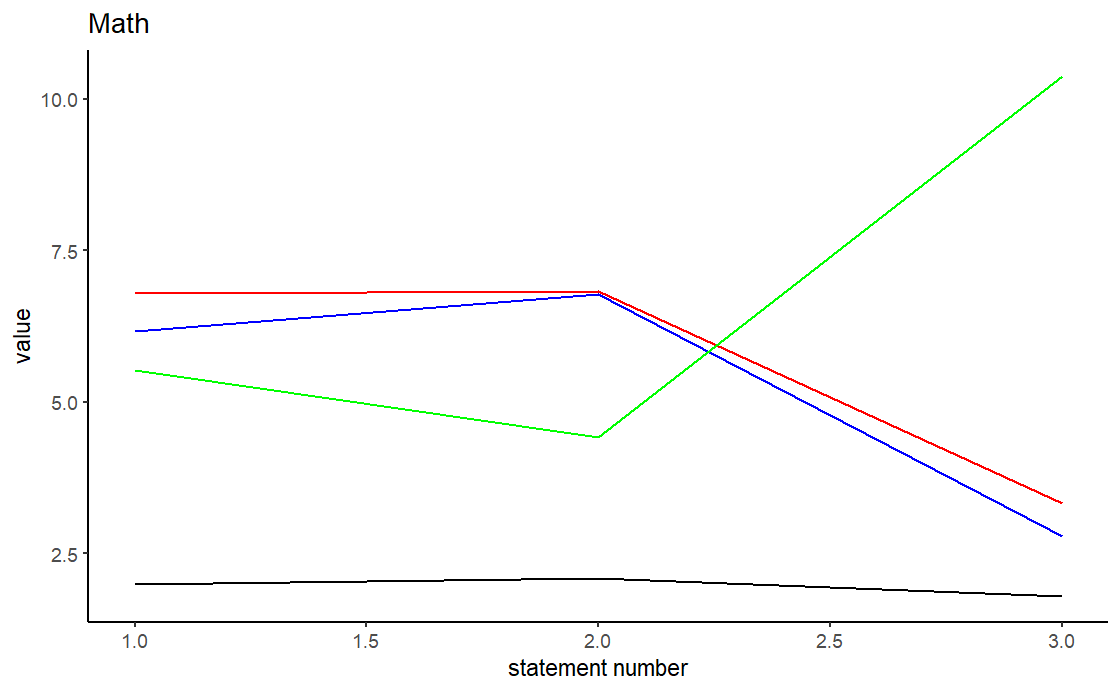


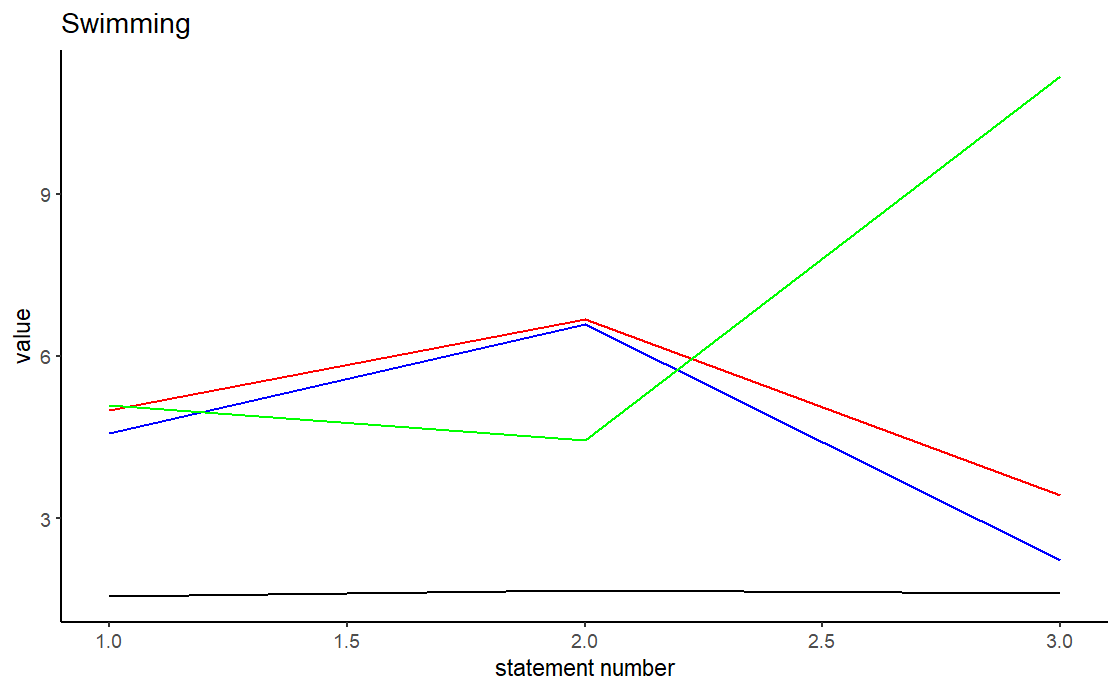


*Note.* The trend for average ratings for each explanation type are represented in a different line color. Green=True, Red/Blue=Lure, Black=Absurd. Scenario names are given at the top-left of each panel. Figures for the whole sample can be found in the supplements of Gadassi-Polack, Bronstein, et al. (2023).
